# The topology of plasminogen binding and activation on the surface of human breast cancer cells

**DOI:** 10.1054/bjoc.2001.2022

**Published:** 2001-09

**Authors:** N M Andronicos, M Ranson

**Affiliations:** Department of Biological Sciences, University of Wollongong, NSW 2522, Australia

**Keywords:** plasminogen binding, co-localisation, urokinase, breast cancer cells

## Abstract

The urokinase-dependent activation of plasminogen by breast cancer cells plays an important role in metastasis. We have previously shown that the metastatic breast cancer cell line MDA-MB-231 over-expresses urokinase and binds and efficiently activates plasminogen at the cell surface compared to non-metastatic cells. The aim of this study was to further characterise plasminogen binding and determine the topology of cell surface-bound plasminogen in terms of its potential for activation. The lysine-dependent binding of plasminogen at 4°C to MDA-MB-231 cells was stable and resulted in an activation-susceptible conformation of plasminogen. Topologically, a fraction of bound plasminogen was co-localised with urokinase on the surfaces of MDA-MB-231 cells where it could be activated to plasmin. At 37°C plasmin was rapidly lost from the cell surface. Apart from actin, other candidate plasminogen receptors were either not expressed or did not co-localise with plasminogen at the cell surface. Thus, based on co-localisation with urokinase, plasminogen binding is partitioned into two functional pools on the surface of MDA-MB-231 cells. In conclusion, these results shed further light on the functional organisation of the plasminogen activation cascade on the surface of a metastatic cancer cell. © 2001 Cancer Research Campaignhttp://www.bjcancer.com


*British Journal of Cancer* (2001) **85**(6), 909–916
© 2001 Cancer Research Campaign
doi: 10.1054/ bjoc.2001.2022, available online at http://www.idealibrary.com on IDEAL

http://www.bjcancer.com

### The topology of plasminogen binding and activation on the surface of human breast cancer cells

**NM Andronicos and M Ranson**

Department of Biological Sciences, University of Wollongong, NSW Australia, 2522

**Summary** The urokinase-dependent activation of plasminogen by breast cancer cells plays an important role in metastasis. We have previously shown that the metastatic breast cancer cell line MDA-MB-231 over-expresses urokinase and binds and efficiently activates plasminogen at the cell surface compared to non-metastatic cells. The aim of this study was to further characterise plasminogen binding and determine the topology of cell surface-bound plasminogen in terms of its potential for activation. The lysine-dependent binding of plasminogen at 4˚C to MDA-MB-231 cells was stable and resulted in an activation-susceptible conformation of plasminogen. Topologically, a fraction of bound plasminogen was co-localised with urokinase on the surfaces of MDA-MB-231 cells where it could be activated to plasmin. At 37˚C plasmin was rapidly lost from the cell surface. Apart from actin, other candidate plasminogen receptors were either not expressed or did not co-localise with plasminogen at the cell surface. Thus, based on co-localisation with urokinase, plasminogen binding is partitioned into two functional pools on the surface of MDA-MB-231 cells. In conclusion, these results shed further light on the functional organisation of the plasminogen activation cascade on the surface of a metastatic cancer cell. © 2001 Cancer Research Campaign http://www.bjcancer.com

**Keywords**: plasminogen binding; co-localisation; urokinase; breast cancer cells

Overexpression of the components of the plasminogen activation cascade such as urokinase plasminogen activator (uPA) are associated with a poor prognosis and a short disease-free period in patients suffering from breast cancer (reviewed in Duffy et al, 1999; Schmitt et al, 2000). Studies of the plasminogen activation cascade using human breast cancer cell lines have revealed insights into this cascade. The metastatic human breast cancer cell line MDA-MB-231 has a high glu-plasminogen binding capacity and cell surface uPA antigen levels (Ranson et al, 1998). This results in the generation of significant amounts of cell-surface-associated plasmin (Ranson et al, 1998) which facilitates the degradation of human endothelial basement membranes (Stonelake et al, 1997).

Glu-plasminogen binds in a lysine-dependent manner to cells with low affinity but high capacity (Felez, 1998). Glu-plasminogen binding to cells is decreased after treatment of the cells with carboxypeptidase B suggesting that a component of binding is C-terminal lysine-dependent (Redlitz et al, 1995a). The low affinity of glu-plasminogen binding has led to suggestions that binding is not important for activation. However, the fact remains that glu-plasminogen activation is enhanced in the presence of small lysine analogues, cells and proteins and that this is attributable to a more open conformation of glu-plasminogen (Markus, 1996).

Multiple proteins with glu-plasminogen binding activity have previously been detected by ligand blots in individual breast cancer cell lines (Ranson et al, 1998). A heterogeneous group of cellular plasminogen receptors has been identified and includes; actin, annexin II, cytokeratin 8, α-enolase, and tetranectin (reviewed in Felez, 1998). Of these plasminogen receptor candidates only cytokeratin 8 and α-enolase possess a C-terminal lysine residue. The other candidates may be considered as latent plasminogen receptors that require plasmin modification for glu-plasminogen binding activity. For example, the glu-plasminogen binding capacity of actin is increased after plasmin modification (Lind and Smith, 1993). Whilst actin may account for approximately 50% of the total glu-plasminogen binding capacity of endothelial cells (Dudani and Gantz, 1996), α-enolase represents only approximately 10% of the total glu-plasminogen binding capacity of U937 cells (Redlitz et al, 1995b).

In contrast to glu-plasminogen binding, single- and twin-chain uPA (sc- and tc-uPA) bind with high affinity to the surfaces of different cell types (such as cancer cells) via a single receptor designated as uPAR (Vassalli et al, 1985; Cubellis et al, 1986). Ellis et al (1999) have demonstrated that glu-plasminogen binds directly to active site blocked uPA in vitro in a lysine-dependent manner ($K_d$ = 50 nM). Non-active site peptides of uPA containing internal lysine residues competitively inhibited both glu-plasminogen cell-surface binding and activation (Ellis et al, 1999). Taken together, this indirectly suggests that cell surface uPA can bind glu-plasminogen via a non-active site interaction and that such binding is necessary for cell-surface activation.

The efficient activation of glu-plasminogen requires both an activation-susceptible conformation of glu-plasminogen (via binding) and presence of plasminogen activators. Clearly, a range of plasminogen receptors, including uPA, may singly or in combination define the total plasminogen binding capacity of a cell. In this study we have shown that glu-plasminogen binds in a stable lysine-dependent manner to breast cancer cells and this results in a conformational change to the zymogen. Furthermore, we have characterised the topology of cell surface plasminogen activation cascade components (glu-plasminogen, uPA, and candidate receptors) responsible for plasmin generation.

*Received 20 October 2000*
*Revised 5 July 2001*
*Accepted 6 July 2001*

*Correspondence to:* M Ranson

### MATERIAL AND METHODS

### Materials

Goat anti-human annexin II polyclonal antibody and goat anti-human actin polyclonal antibody were purchased from Santa Cruz Biotechnology Inc; both antibodies were raised against C-terminal peptides of their antigens. Mouse anti-human cytokeratin 8 monoclonal antibody was purchased from Novocastra Inc. Mouse anti-human uPA monoclonal antibody (#394; recognises all forms of human uPA including receptor-bound uPA and B-chain fragment/active mutants) and mouse anti-human uPAR monoclonal antibody (#3936) were purchased from American Diagnostica Inc (USA). FITC conjugated sheep anti-goat IgG, Cy3-conjugated sheep anti-goat IgG, Cy3-conjugated goat anti-mouse IgG, propidium iodide (PI) and tranexamic acid (TA) were purchased from Sigma Chemical Co. FITC-conjugated goat anti-mouse IgG was purchased from Silenus (Australia). Glu-gly-arg chloromethylketone (EGR-CMK) was purchased from Calbiochem (Australia). Rabbit polyclonal antibody to human recombinant α-enolase was prepared as described (Andronicos et al, 1997). These antibodies recognise cellular α-enolase in whole cell lysates (Andronicos et al, 1997) and in fixed, permeabilised breast cancer cell lines (data not shown). Cy3 and Cy5 were purchased from Amersham Pharmacia Biotech (Australia).

### Purification and labelling of proteins

Glu-plasminogen was purified using lysine Sepharose 4B as described (Andronicos et al, 1997) and FITC-conjugated as described (Ranson et al, 1998) or Cy5-conjugated according to the manufacturer's instructions. The lysine-binding capacity of the fluorochrome-conjugated glu-plasminogen was verified by lysine Sepharose affinity chromatography and SDS-PAGE (data not shown). Bovine aprotinin (Roche Diagnostics) was conjugated with FITC or Cy5 as described above. Unlabelled and Cy5- or FITC-labelled aprotinin inhibited plasmin-mediated fibrinogen proteolysis to similar extents (data not shown). All conjugated proteins were aliquoted and stored at –70˚C until required.

### Cell culture

The MCF-7 and MDA-MB-231 human breast cancer cell lines were routinely cultured as previously described (Ranson et al, 1998). For all experiments cells were cultured for approximately 48 h in either flasks or on coverslips without a change of media. Unless otherwise specified, all experiments were performed using MDA-MB-231 cells.

### Flow cytometry and fluorimetry

Both glu-plasminogen binding and immunocytometry assays were performed using dual colour flow cytometry with propidium iodide (PI) to distinguish between viable and non-viable cells as previously described (Ranson et al, 1998). The stability of glu-plasminogen or plasmin activity on the surfaces of viable MDA-MB-231 cells at 37˚C was determined using a modification of the dual colour flow cytometry method. Briefly, cells were incubated with either FITC-glu-plasminogen or glu-plasminogen at 4˚C, washed, and resuspended in binding buffer pre-warmed to 37˚C for various times. At specific times up to 10 min the reactions were terminated by adding 50 μg $ml^{-1}$ of either aprotinin or FITC-aprotinin and analysed immediately by dual colour flow cytometry.

A plasminogen conformational change assay was developed whereby cells ($1 \times 10^6$ $ml^{-1}$) were incubated in black 96-well fluorescence plates (Costar) with FITC-glu-plasminogen (50 μg $ml^{-1}$) in the absence or presence of the tc-uPA specific inhibitor EGR-CMK (0.5 mM) for 90 min on ice in the dark. The total fluorescence of each well was measured at 5 min intervals using a Biolumin 960 plate reader equipped with a FITC 480/520 excitation/emission filter set.

### Confocal microscopy

Fixation and quenching of the cells was performed according to the method of Bastiaens and Jovin (1996). The fixed and quenched samples were washed twice with PBS, blocked for 1 h with PBS/0.1% BSA at room temperature and then washed once with ice-cold PBS/0.1% BSA. The samples were incubated for 1 h on ice and in the dark with 200 μl of PBS/0.1% BSA supplemented with 50 μg $ml^{-1}$ Cy5-plasminogen. After three 5 min incubations with 1 ml of ice-cold PBS/0.1% BSA, the samples were incubated with 200 μl of PBS/0.1% BSA containing either 20 μg $ml^{-1}$ of a Cy3-labelled antibody (FRET analysis) or an unlabelled antibody for 30 min on ice and then washed as described above. If an unlabelled antigen-specific primary antibody was used then the samples were subsequently incubated on ice with a 1:50 dilution of a Cy3 secondary antibody for 30 minutes and washed before mounting. The coverslips were mounted onto microscope slides using 2 mm wide plastic spacers and 5 μl of PBS containing 20% (v/v) glycerol as the mounting medium.

Fluorescence resonance energy transfer (FRET) experiments were performed as described by Bastiaens and Jovin (1996). However, microinjection of labelled protein and permeabilisation of cells were not required because FRET was used to determine the proximity of cell surface antigens.

To determine cell-surface plasmin activity, adherent cells were washed twice with ice-cold PBS/0.1% BSA and then incubated on ice for 1 h with 200 μl PBS/0.1% BSA containing 50 μg $ml^{-1}$ of glu-plasminogen. After two 5 min washes with PBS/0.1% BSA, the samples were incubated at 10˚C for 5 min in 200 μl PBS/0.1% BSA. This relatively gentle activation step was necessary to prevent the cells detaching from their substrata. The reaction was stopped by adding Cy5-aprotinin (1 μM) and incubating for a further 30 min on ice in the dark. After two 5 min washes with PBS/0.1%BSA, the samples were fixed and quenched as described above. The samples were subsequently incubated with uPA monoclonal antibody (#394) and the sheep anti-mouse IgG Cy3 secondary antibody as described above. Finally the cells were mounted and examined by confocal microscopy.

All confocal microscopy experiments were performed a minimum of two times and images from a single representative experiment are shown. All confocal laser scanning microscopy images were acquired using a Leica TCS SP system fitted with 1.3 NA oil immersion objective lenses. The Cy3 and Cy5 fluorophores were excited with the 543 nm and 633 nm spectral lines of a He-Ne laser. The emissions of Cy3 and Cy5 were collected between 560–590 nm and greater than 650 nm, respectively. All images were analysed using TCS NT software, version 1.6.587 (Leica, Heidlberg, Germany).

 © 2001 Cancer Research Campaign

### Statistical analysis

Statistical comparisons of the data were made using Student's *t*-test (two-sample assuming unequal variances).

### RESULTS

### Stability and conformation of cell bound plasminogen

In a previous study we found that even though glu-plasminogen binds to MDA-MB-231 cells with low affinity but high capacity, the interaction appeared to be stable under conditions that minimised activation (Ranson et al, 1998). To examine the lysine-dependent stability of glu-plasminogen binding to MDA-MB-231 cell surfaces the TA-dependent association and dissociation phases of glu-plasminogen binding were compared (Figure 1A). Half-maximal inhibition of glu-plasminogen association was achieved with approximately 50 μM TA, while half-maximal dissociation of bound glu-plasminogen was achieved with approximately 250 μM TA. Furthermore, approximately 25% of bound glu-plasminogen could not be stripped from the cells, even with 200 mM TA (Figure 1A). In contrast, this concentration of TA competitively inhibited 90% of glu-plasminogen binding. Since glu-plasminogen was more resistant to TA dissociation than association, this suggested that a lysine-dependent interaction at the cell surface stabilised the binding of glu-plasminogen.

Lysine-dependent conformational differences between free and cell-bound glu-plasminogen may contribute to the stability of cell surface-associated glu-plasminogen. Small changes in the total fluorescence of glu-plasminogen (typically 10%) in the presence of lysine analogues or proteins are representative of lysine-dependent conformational changes to the zymogen (Christensen and Molgaard, 1991; Andronicos et al, 2000). Similarly, the change in total fluorescence of FITC-glu-plasminogen in the presence of MDA-MB-231 cells increased to a maximum of approximately 10% in a time-dependent manner (Figure 1B). Furthermore, the presence of the uPA inhibitor EGR-CMK did not significantly alter the change in total fluorescence of glu-plasminogen ($P > 0.25$; Figure 1B), suggesting that the conformational change was independent of endogenous uPA activity. Taken together, these results suggest that the binding of glu-plasminogen to the cell surface may induce a conformational change, which may affect the stability of bound glu-plasminogen.

When activation of cell-bound FITC-glu-plasminogen at 37°C was terminated at different time points with aprotinin, a maximum of 40% of the total cell-associated fluorescence was lost from the surface of MDA-MB-231 cells (Figure 2A). When activation of cell-bound glu-plasminogen at 37°C was terminated at different time points with FITC-aprotinin, a technique that allows the simultaneous inhibition and detection of plasmin (Ranson et al, 1998; O'Mullane and Baker, 1998), approximately 75% of cell-surface plasmin was lost by 20 min (Figure 2B). In contrast, when activation was terminated after 5 min with FITC-aprotinin and cells sampled at various time points after inhibition, no further loss of plasmin occurred, even after several hours (data not shown). Thus, it appeared that at 37°C the turnover of cell surface plasmin(ogen) was due to proteolysis rather than a non-proteolytic, dissociation event.

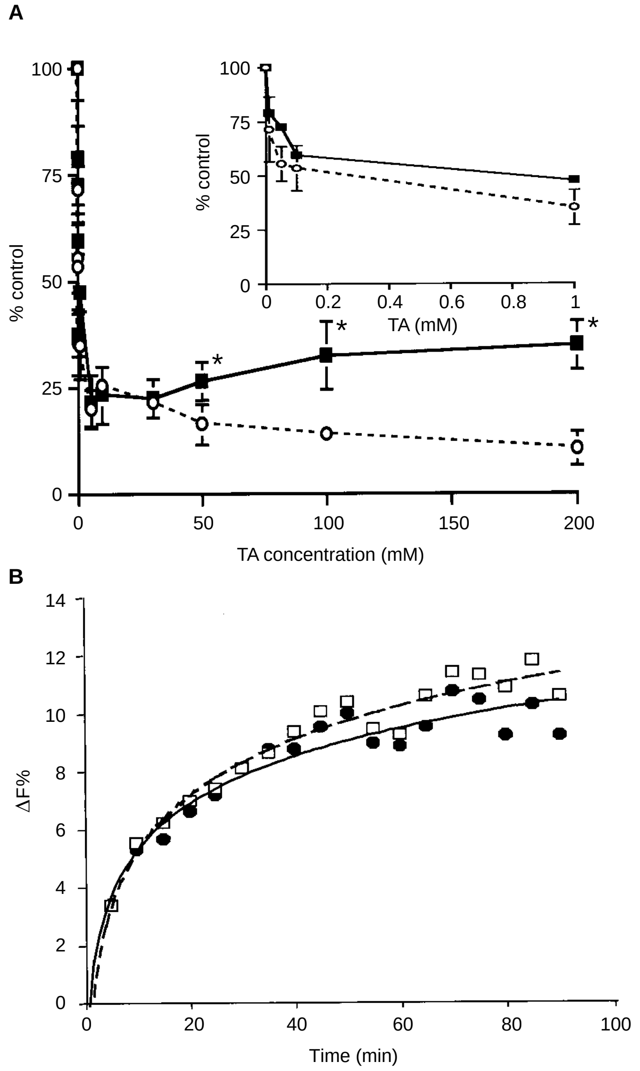

**Figure 1** (**A**) TA competition curves of glu-plasminogen binding to the MDA-MB-231 cell surface. (○) The association phase competitive inhibition of FITC-glu-plasminogen (50 μg ml$^{-1}$) binding to MDA-MB-231 cells in the presence of increasing concentrations of TA. (■) Competitive dissociation of FITC-glu-plasminogen bound to MDA-MB-231 cells. Cells were loaded with FITC-glu-plasminogen (50 μg ml$^{-1}$), washed and the bound FITC-glu-plasminogen dissociated with increasing concentrations of TA. Inset: the association and dissociation curves for TA concentrations up to 1 mM TA. Values shown are means ± SEM from 3 separate experiments. *Significantly different from association (% control) at corresponding concentrations of TA ($P < 0.05$). (**B**) Time course of the change in total fluorescence of FITC-glu-plasminogen induced by MDA-MB-231 cells at 4°C in either the presence (●) or absence (□) of EGR-CMK. Both curves fitted logarithmic expressions (●; $r^2 = 0.91$: □; $r^2 = 0.95$). Values shown are data from a representative experiment

### Candidate cell-surface plasminogen receptor expression

The MCF-7 and MDA-MB-231 cell-surface expression levels of known plasminogen receptor candidates, for which antibodies were available to us, were examined by dual-colour flow cytometry (Figure 3A). Both of the preformed plasminogen receptors, α-enolase and cytokeratin 8 antigens, were not detected on the surfaces of either cell line (Figure 3A). Notably, while both cell lines expressed comparable levels of cell-surface annexin II ($P = 0.32$), MCF-7 cells expressed significantly more (4.5 fold) cell-surface actin ($P = 0.019$), than did the MDA-MB-231 cells. Immunohistochemical analysis of fixed, non-permeabilised MDA-MB-231 and MCF-7 cells visually confirmed these findings (data

© 2001 Cancer Research Campaign

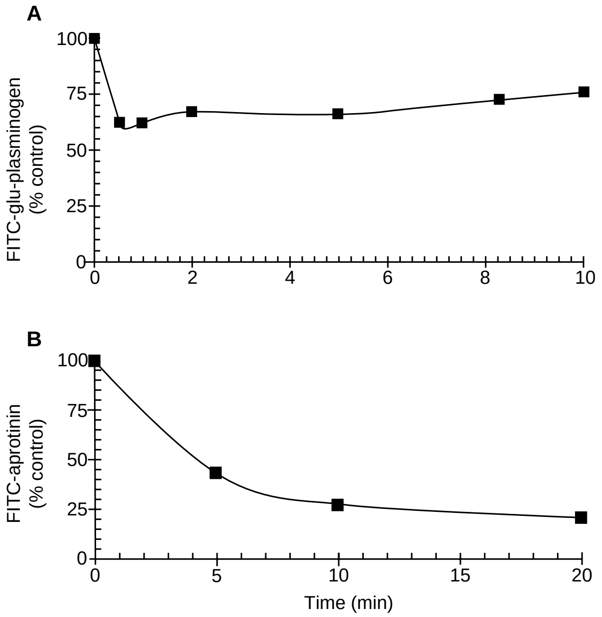

**Figure 2** Loss of bound glu-plasminogen (**A**) and plasmin activity (**B**) from the surfaces of viable MDA-MB-231 cells incubated at 37°C. Cells were incubated with 50 μg $ml^{-1}$ of either FITC-glu-plasminogen (**A**) or glu-plasminogen (**B**) for 1 h on ice, washed and incubated at 37°C for various times. The reactions were terminated with 50 μg $ml^{-1}$ of either aprotinin (**A**) or FITC-aprotinin (**B**). All samples were acquired by dual colour flow cytometry and only PI negative (viable) cell populations were analysed. Values shown are means of duplicate determinations from a representative experiment not shown). Thus, the high and low glu-plasminogen-binding capacities of the MDA-MB-231 and MCF-7 cell lines, respectively, could not be explained simply by the cell-surface expression levels of annexin II or actin.

### Relationship between cell-surface uPA expression and plasminogen-binding capacity

The high plasminogen-binding MDA-MB-231 cell line expressed approximately 40- and 20-fold more cell-surface uPA and uPAR, respectively, than the non-metastatic, low-plasminogen-binding MCF-7 cell line (Figure 3B). As a direct, non-active site interaction between glu-plasminogen and uPA has been demonstrated by surface plasmon resonance (Ellis et al, 1999), it seemed possible that uPA may contribute to the total glu-plasminogen-binding capacity of the MDA-MB-231 cells.

To investigate this relationship, the distribution of uPA and glu-plasminogen were determined by confocal microscopy on fixed non-permeabilised, adherent MDA-MB-231 cells (Figure 4). MDA-MB-231 cells were incubated with Cy5-glu-plasminogen (red), then with an anti-uPA monoclonal antibody and Cy3-conjugated secondary antibody (green). Urokinase antigen showed a punctate distribution on the surfaces of the cells (Figure 4D). In contrast, Cy5-glu-plasminogen was diffusely distributed over the cells, with areas of high local concentrations at the extremities of most cells (Figure 4E). As previously shown by ligand histochemistry (Ranson et al, 1998), Cy5-glu-plasminogen binding was also lysine-dependent since competition with 5 mM TA inhibited plasminogen binding (data not shown). An overlay of the Cy3-uPA antigen (green) and Cy5-glu-plasminogen (red) images resulted in defined regions of red and green as well as regions of intense yellow fluorescence signals on the cell surfaces (Figure 4F). The presence of yellow fluorescence suggested that glu-plasminogen and uPA were co-localised at these regions. A three-dimensional reconstruction of two adherent MDA-MB-231 cells (Figure 4G) demonstrated that the distribution of co-localised glu-plasminogen and uPA was on a lateral surface of the cell.

Bound Cy5-glu-plasminogen did not prevent the uPA monoclonal antibody binding to its B-chain epitope (Figure 4), and pre-incubation with 50 μg $ml^{-1}$ of this antibody did not significantly inhibit the glu-plasminogen binding capacity of MDA-MB-231 cells as assessed by flow cytometry (data not shown). Therefore, while co-localisation may be due to a direct interaction between glu-plasminogen and uPA on the MDA-MB-231 cells, it was unlikely to be via the active site of uPA. Moreover, maximal plasmin activity (as detected by Cy5-aprotinin after activation of bound plasminogen) corresponded to regions of high uPA antigen concentrations on these cells (data not shown), further suggesting that a fraction of glu-plasminogen may bind to uPA in a non-active site manner whereupon it can be activated to plasmin at the cell surface.

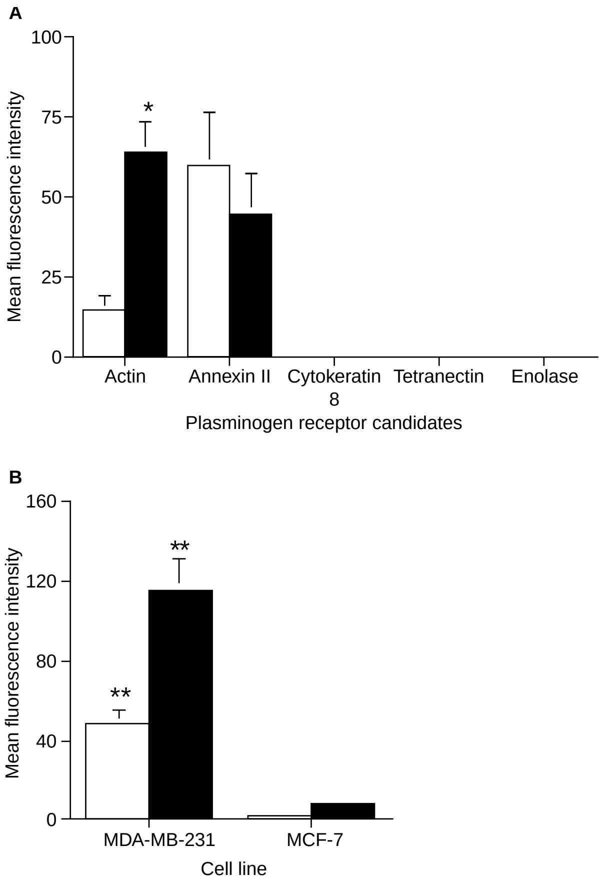

**Figure 3** Expression of known components of the plasminogen activation cascade on the surfaces of viable MDA-MB-231 and MCF-7 cells. (**A**) MDA-MB-231 (□) and MCF-7 (■) cells were probed with antibodies specific for different known plasminogen receptor candidates or a non-specific primary antibody and analysed by dual-colour flow cytometry. Non-specific cell-surface fluorescence was subtracted from all values. Bar heights indicate means ± SEM of 3 separate experiments. *Significantly different from actin levels on MDA-MB-231 cells ($P < 0.05$). (**B**) MDA-MB-231 or MCF-7 cells were probed with 20 μg $ml^{-1}$ of anti-uPA (□), anti-uPAR (■) monoclonal antibodies or their corresponding isotype control antibodies and analysed by dual-colour flow cytometry. Isotype-associated fluorescence was subtracted from all values. Bar heights indicate means ± SEM of 3 separate experiments. ** Significantly different from values associated with MCF-7 cells ($P < 0.021$)

 © 2001 Cancer Research Campaign

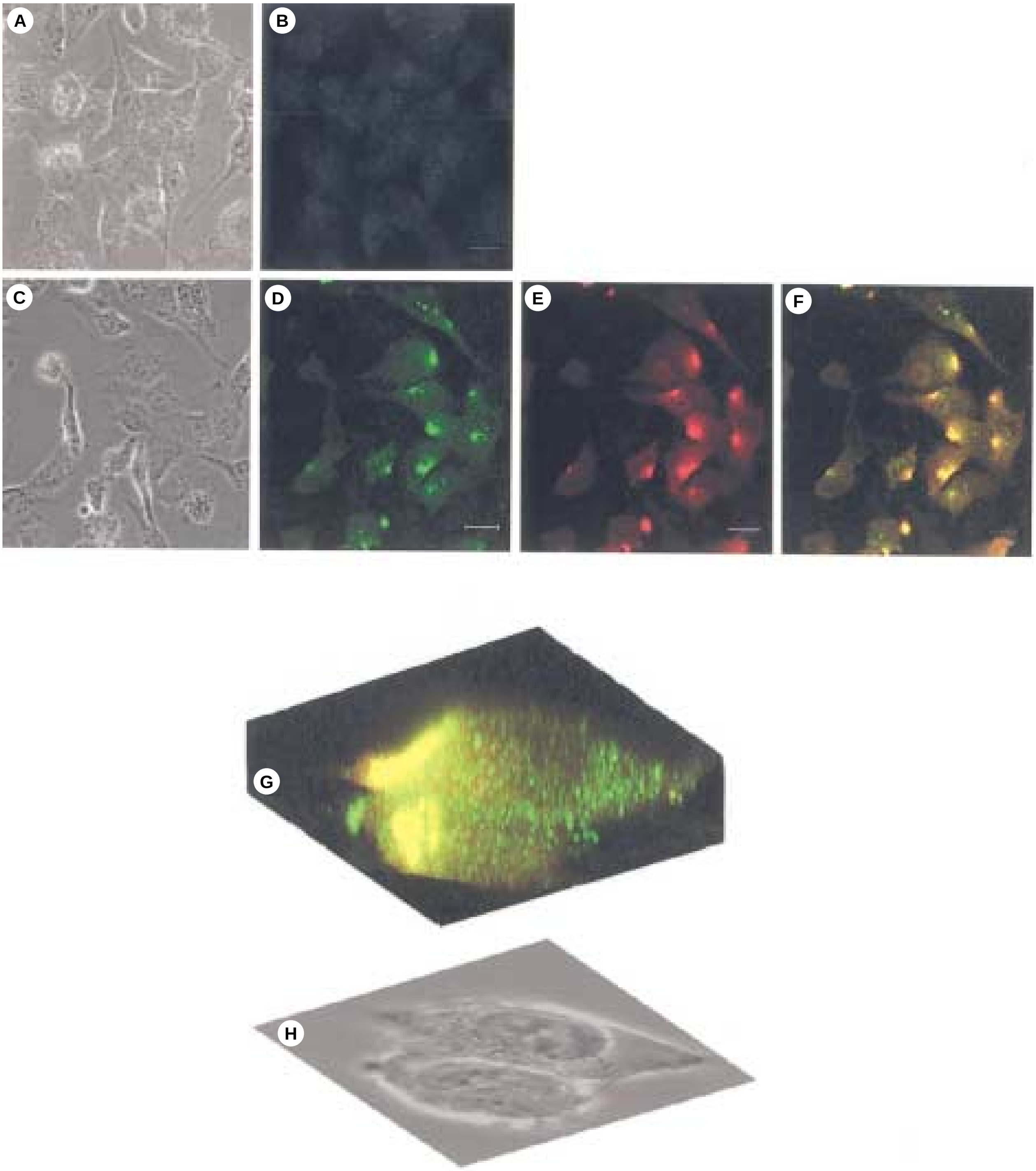

**Figure 4** The co-distribution of uPA and glu-plasminogen on the surfaces of adherent, non-permeabilised MDA-MB-231 cells by confocal microscopy. (**A**, **C**, and **H**) Transmission images. (**B**) $IgG_1$ isotype control antibody. (**D** and **E**) The distribution of Cy3-uPA antigen (green) and Cy5-glu-plasminogen (red), respectively. (**F**) An overlay of **D** and **E** demonstrating the distribution of Cy5-glu-plasminogen (red) and Cy3-uPA antigen (green) on surfaces of MDA-MB-231 cells. (**G**) A three dimensional reconstruction of two cells demonstrating the distribution of Cy3-uPA antigen (green) and Cy5-glu-plasminogen (red) on the surfaces of these cells. All fluorescence images are maximum projection composites of at least 8 optical sections. The total depth of the images ranged between 7.3–12.2 μm. Scale bars represent 20 μm

The existence of glu-plasminogen-binding sites (red) that did not co-localise with uPA (green) (Figure 4) indicated that a proportion of the glu-plasminogen bound to the cell surface was due to the presence of other receptor molecules.

### Relationship between cell-surface annexin II and actin expression and plasminogen binding

Confocal microscopy was used to determine whether the cell-surface plasminogen receptor candidates actin and annexin II

co-localised with glu-plasminogen and therefore contribute to the plasminogen-binding capacity of MDA-MB-231 cells (Figure 5). In contrast to glu-plasminogen, annexin II antigen had a punctate distribution on the cell surface (Figure 5D). An overlay of the annexin II antigen (green) and glu-plasminogen (red) images resulted in well defined, non-overlapping regions of red and green without any regions of yellow (overlapping) fluorescence (Figure 5F). This qualitatively suggested that annexin II and glu-plasminogen were not obviously co-localised on the cell surface. The non-overlap of the fluorescence emissions from Cy3-annexin II antibody and Cy5-glu-plasminogen was quantitatively confirmed using fluorescent energy transfer (FRET) analysis. The apparent FRET efficiency for the Cy3-Cy5 system on MDA-MB-231 cell surfaces was approximately 2%, indicating that Cy3-annexin II antibody did not efficiently donate energy to the Cy5-glu-plasminogen acceptor.

The distribution pattern of actin antigen was punctate with regions of high fluorescence at the extremities of the cells (Figure 5H). Most cells contained at least one area of cell-surface actin-glu-plasminogen co-localisation (Figure 5J; yellow fluorescence). A three-dimensional reconstruction of optical sections of a cell probed with Cy5-glu-plasminogen and an actin antibody detected with Cy3-conjugated secondary antibody demonstrated that glu-plasminogen was co-localised with actin antigen on a lateral surface of the cell membrane (Figure 5M). Since MCF-7 cells express significantly more cell-surface actin but bind significantly less glu-plasminogen than MDA-MB-231 cells, we investigated their distribution patterns on fixed, non-permeabilised, adherent MCF-7 cells. In contrast to MDA-MB-231 cells, there was no co-localisation (i.e. yellow fluorescence) on the majority of MCF-7 cells (data not shown). Taken together, these data suggest that actin may also be responsible for binding a fraction of the total surface localised glu-plasminogen on MDA-MB-231 cells but not on MCF-7 cells.

### DISCUSSION

The efficient activation of glu-plasminogen requires both the activation-susceptible conformation of zymogen and presence of plasminogen activators. The conformational change of glu-plasminogen can be mediated by small lysine analogues and α-enolase (Markus, 1996; Andronicos et al, 2000). Whilst the binding of glu-plasminogen to cells is low affinity (Felez, 1998; Ranson et al, 1998) this study demonstrated that binding may also stabilise an activation-susceptible conformation of glu-plasminogen on the cell surface. Furthermore, this study mapped the topology of cell surface-bound glu-plasminogen in terms of its potential for activation. The results suggest that the fraction of cell-surface-bound glu-plasminogen that co-localises with uPA may represent the pool of cell-surface plasminogen that is readily activated.

The stability of binding on the cell surface was demonstrated by the fact that it was easier to prevent binding than it was to strip bound glu-plasminogen with TA. This was accompanied by an increase in the total fluorescence of FITC-glu-plasminogen (in the presence or absence of EGR-CMK), suggesting that binding changes the conformation of glu-plasminogen from the closed to the more open isomer independent of tc-uPA activity. Thus, MDA-MB-231 cells may promote the activation of glu-plasminogen by binding it in a stable, activation-susceptible conformation.

The second requirement for efficient activation of glu-plasminogen is access to plasminogen activators. Ellis et al (1999) previously demonstrated that glu-plasminogen binds to a lysine residue on the A-chain of cell-surface uPA. In the current study, bound glu-plasminogen had a uniform cell-surface distribution whereas the endogenous uPA distribution was punctate. Defined regions of yellow fluorescence indicated that a proportion of glu-plasminogen that bound to the MDA-MB-231 cells co-localised with uPA on the lateral surfaces of these cells. Furthermore, the uPA/aprotinin (plasmin) distribution patterns suggested that uPA co-localised glu-plasminogen can be activated at the cell surface. The anti-uPA antibody used in this study is directed against the B-chain of uPA and did not inhibit glu-plasminogen binding to the MDA-MB-231 cells. This suggests that if glu-plasminogen does bind to uPA directly, then it must be via the A-2 chain and not an active site-dependent interaction. This would support the indirect evidence presented by Ellis et al (1999) that glu-plasminogen binds in a lysine-dependent manner to cell-surface uPA. We are currently raising antibodies to the peptide corresponding to the glu-plasminogen-binding site of uPA (Ellis et al 1999) to analyse the contribution of uPA to the total glu-plasminogen binding capacity of the MDA-MB-231 cells. Commercially available antibodies raised against the A-chain of uPA have been designed to block binding to uPAR and would not be useful for the above purpose.

At 37˚C both cell-surface-bound glu-plasminogen and plasmin were rapidly turned over. However, the fact that only 40% of glu-plasminogen was lost from the cell surface confirms the topological data which show that only a fraction of glu-plasminogen co-localises with uPA where it is activated to plasmin. Once generated 75% of the plasmin activity was rapidly lost from the surfaces of viable MDA-MB-231 cells, probably due to plasmin proteolysis.

Multiple proteins capable of binding glu-plasminogen have been observed in plasma membrane preparations of MDA-MB-231 cells (Ranson et al, 1998). These findings as well as the high number of binding sites for glu-plasminogen per cell (Ranson et al, 1998) suggest that multiple proteins are responsible for localising glu-plasminogen to the cell surface. The plasminogen receptors, actin and annexin II were present on the surfaces of the MDA-MB-231 cells. However, only actin was co-localised with glu-plasminogen suggesting that actin may function as a cell-surface plasminogen-binding protein. Urokinase and actin antigens were also co-localised on the lateral surfaces of MDA-MB-231 cells (data not shown). This raises the possibility of a ternary complex between glu-plasminogen, actin and uPA on the lateral surfaces of viable cells to facilitate plasmin generation.

Cell-surface-bound glu-plasminogen exists in two distinct functional pools (activatable and non-activatable) in vitro. The presence of glu-plasminogen in a non-activatable pool does not preclude the possibility of activation by stromal plasminogen activators in vivo (Dano et al, 1999). However, the fact remains that high tumour cell uPA expression correlates with poor prognosis (reviewed in Duffy et al, 1999; Schmitt et al, 2000). In conclusion, we have defined three criteria critical for cell-surface glu-plasminogen activation: (1) glu-plasminogen binding, (2) lysine-dependent conformational change of glu-plasminogen to an activation-susceptible form, and (3) co-localisation with uPA. Satisfaction of all these criteria on the surface of MDA-MB-231 cells facilitates efficient plasmin-mediated pericellular proteolysis, which in turn contributes to the invasive and metastatic capacity of these cells.

© 2001 Cancer Research Campaign

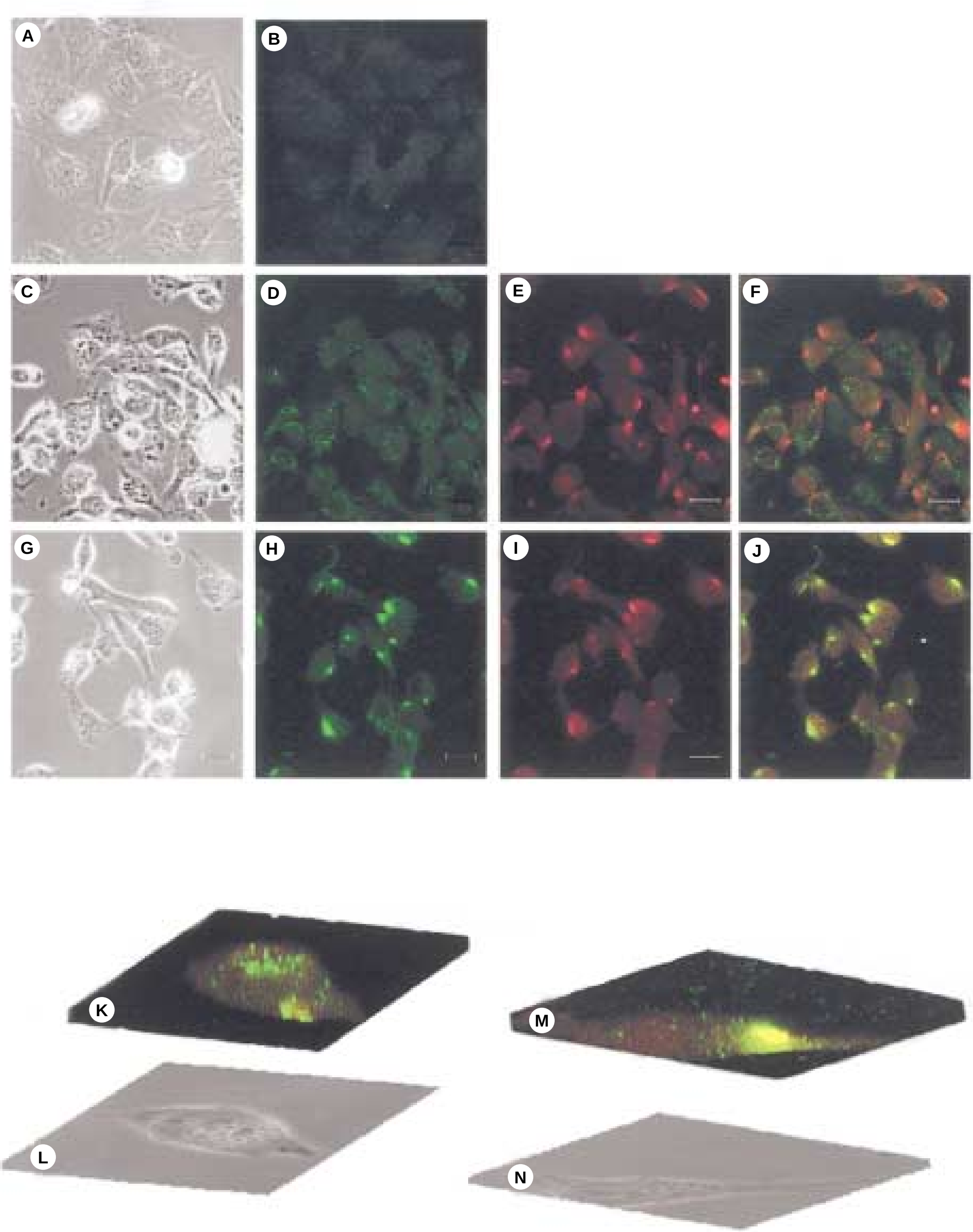

**Figure 5** The co-distribution of glu-plasminogen with annexin II or actin on the surfaces of adherent, non-permeabilised MDA-MB-231 cells by confocal microscopy. (**A**, **C**, **G**, **L** and **N**) Transmission images. (**B**) Purified, normal goat IgG negative control antibodies. (**D** and **E**) The distribution of Cy3-annexin II antigen (green) and Cy5-glu-plasminogen (red), respectively. (**F**) An overlay of **D** and **E** demonstrating the distribution of both Cy5-glu-plasminogen and Cy3-annexin II antigen. (**H** and **I**) The distribution of Cy3-actin antigen (green) and Cy5-glu-plasminogen (red), respectively. (**J**) An overlay of **H** and **I** demonstrating the distribution of both Cy5-glu-plasminogen and Cy3-actin antigen. Three-dimensional reconstructions of MDA-MB-231 cells showing the distribution of either Cy3-annexin II antigen and Cy5-glu-plasminogen (**K**) or Cy3-actin antigen and Cy5-glu-plasminogen (**M**) on the surfaces of the cells. All fluorescence images are maximum projection composites of at least 8 optical sections. The total depth of the images ranged between 6.1–12.2 μm. Scale bars represent 20 μm

*© 2001 Cancer Research Campaign*

### ACKNOWLEDGEMENTS

NMA was funded by a National Health and Medical Research Council of Australia Project Grant #970808.

### REFERENCES

Andronicos NM, Ranson M, Bognacki J and Baker MS (1997) The human ENO1 gene product (recombinant human α-enolase) displays characteristics required for a plasminogen binding protein. *Biochim Biophys Acta* **1337**: 27–39

Andronicos NM, Baker MS, Lackmann M and Ranson M (2000) Deconvolution of the binding of plasminogen to its receptor α-enolase. *Fibrinol Proteol* **14**: 327–336

Bastiaens PIH and Jovin TM (1996) Microspectroscopic tracks the intracellular processing of a signal transduction protein: Fluorescently-labeled protein kinase C β1. *Proc Natl Acad Sci USA* **93**: 8407–8412

Christensen U and Molgaard L (1991) Stopped-flow kinetic studies of glu-plasminogen. Conformational changes triggered by AH-site ligand binding. *FEBS Lett* **278**: 204–206

Cubellis MV, Nolli ML, Cassani G and Blasi F (1986) Binding of single-chain prourokinase to the urokinase receptor of human U937 cells. *J Biol Chem* **261**: 15818–15822

Dano K, Romer J, Nielsen BS, Bjorn S, Pyke C, Rygaard J and Lund LR (1999) Cancer invasion and tissue remodeling-cooperation of protease systems and cell types. *APMIS* **107**: 120–127

Dudani AK and Ganz PR (1996) Endothelial cell surface actin serves as a plasminogen binding site for plasminogen, tissue plasminogen activator and lipoprotein (a). *Br J Haematol* **95**: 168–178

Duffy MJ, Maguire TM, McDermott EW and O' Higgins N (1999) Urokinase plasminogen activator: a prognostic marker in multiple types of cancer. *J Surg Oncol* **71**: 130–135

Ellis V, Whawell SA, Werner F and Deadman JJ (1999) Assembly of urokinase-receptor-mediated plasminogen activation complexes involves direct, non-active-site interactions between urokinase and plasminogen. *Biochemistry* **38**: 651–659

Felez J (1998) Plasminogen binding to cell surfaces. *Fibrinol Proteol* **12**: 183–189

Lind SE and Smith CJ (1993) Actin stimulates plasmin generation by tissue and urokinase-type plasminogen activators. *Arch Biochem Biophys* **307**: 138–145

Markus G (1996) Conformational changes in plasminogen, their effect on activation and agents that modulate activation rates – a review. *Fibrinolysis* **10**: 75–85

O'Mullane MJ and Baker MS (1998) Loss of cell viability dramatically elevates cell-surface plasminogen binding and activation. *Expt Cell Res* **242**: 153–164

Ranson M, Andronicos NM, O' Mullane MJ and Baker MS (1998) Increased plasminogen binding is associated with metastatic breast cancer cells: differential expression of plasminogen binding proteins. *Br J Cancer* **77**: 1586–1597

Redlitz A, Tan AK, Eaton DL and Plow EF (1995a) Plasma carboxypeptidases as regulators of the plasminogen system. *J Clin Invest* **96**: 2534–2538

Redlitz A, Fowler BJ, Plow EF and Miles LA (1995b) The role of an enolase-related molecule in plasminogen binding to cells. *Eur J Biochem* **227**: 407–415

Schmitt M, Wilhelm OG, Reuning U, Kruger A, Harbeck N, Lengyel E, Graeff H, Gansbacher B, Kessler H, Burgle M, Sturzebecher J, Sperl S and Magdolen V (2000) The urokinase plasminogen activation system as a novel target for tumour therapy. *Fibrinol & Proteol* **14**: 114–132

Stonelake PS, Jones CE, Neoptolemos JP and Baker PR (1997) Proteinase inhibitors reduce basement membrane degradation by human breast cancer cells. *Br J Cancer* **75**: 951–959

Vassalli J-D, Baccino D and Berlin D (1985) A cellular binding site for the Mr 55,000 form of the human plasminogen activator, urokinase. *J Cell Biol* **100**: 86–92

 *© 2001 Cancer Research Campaign*
